# Advances in Transdermal Delivery Systems for Treating Androgenetic Alopecia

**DOI:** 10.3390/pharmaceutics17080984

**Published:** 2025-07-30

**Authors:** Shilong Xu, Lian Zhou, Haodong Zhao, Siwen Li

**Affiliations:** 1 State Key Laboratory of Natural Medicines, Department of Biomedical Engineering, School of Engineering, China Pharmaceutical University, No. 639 Longmian Avenue, Jiangning District, Nanjing 211198, China; 3323081994@stu.cpu.edu.cn (S.X.); zlttkx2022@163.com (L.Z.); 2 NMPA Key Laboratory for Impurity Profile of Chemical Drugs, Jiangsu Institute for Drug Control, Nanjing 210019, China; zhaohaodong@jsifdc.org.cn

**Keywords:** androgenetic alopecia, transdermal drug delivery system, nanocarriers

## Abstract

Androgenetic alopecia (AGA) is the most prevalent form of alopecia areata. Traditional treatment options, including minoxidil, finasteride, and hair transplantation, have their limitations, such as skin irritation, systemic side effects, invasiveness, and high costs. The transdermal drug delivery system (TDDS) offers an innovative approach for treating AGA by administering medications through the skin to achieve localized and efficient delivery while overcoming the skin barrier. This review systematically explores the application of TDDS in AGA treatment, highlighting emerging technologies such as microneedles (MNs), liposomes, ionic liquids (ILs), nanostructured lipid carriers (NLCs), and transporters (TFs). It analyzes the underlying mechanisms that enhance drug penetration through hair follicles. Finally, this review presents a forward-looking perspective on the future use of TDDS in the management of AGA, aiming to provide insights and references for designing effective transdermal drug delivery systems for this condition.

## 1. Introduction

Androgenetic alopecia (AGA), also known as androgenic alopecia or male pattern baldness, is the most common type of progressive hair loss. Male patients usually present with progressive hair thinning, with the crown and anterior region of the scalp affected and a receding, horseshoe-shaped hairline. Female patients typically present with diffuse alopecia with frontal and crown hair thinning. The condition affects at least 80% of men and 50% of women over the age of 70 and can lead to perception of damage to personal image, with potential for development of more serious social and psychological problems [1,2]. However, its pathogenesis is more complex, involving androgen metabolism and its interaction with androgen receptor, hair follicle growth cycle disorders, and other factors [2]. Currently, the more effective first-line drugs are minoxidil and finasteride [3]. Minoxidil requires long-term use, and frequent topical sprays can cause itching and redness of the skin [4]. The systemic effects of oral finasteride can lead to a range of more serious side effects, including erectile dysfunction and gynecomastia [5]. Surgical treatments represented by hair transplantation can significantly improve patients’ hair density, but their application is limited by invasiveness, long recovery period, and high cost. In addition, medications such as minoxidil must be used continuously for a period of time after surgery to alleviate post-operative telogen effluvium [6]. Therefore, there is an urgent need to develop new safe, effective, and patient-friendly treatment options.

Transdermal drug delivery system (TDDS) is a drug delivery system in which a drug is applied on to the skin surface via a drug-containing device and passes through the skin at a set rate to achieve localized therapy [7,8]. Compared to injectable drug delivery, TDDS administration is simple, cost-effective, non-invasive or minimally invasive, and patient-friendly. Its topical application can circumvent the effects of gastrointestinal pH and digestive enzymes on the active drug, bypass first-pass metabolism, and ultimately improve the bioavailability of the active drug [9,10,11]. Continuous and controlled drug delivery, very low pain, sound economics, and patient compliance [12] make TDDS an excellent choice for the topical delivery of active drugs for treating AGA. TDDS leaves narrow ranges of options for formulators to deliver various active moieties due to its restriction on adherence to selection criteria, including low molecular weight, short half-life, high permeability, optimum oil/water partitioning behavior, and low melting point [13]. The vast majority of active drugs used to treat AGA, such as minoxidil, cannot meet all of these conditions simultaneously. Delivering minoxidil in a simple spray form is extremely inefficient, requiring repeated administration, which can easily cause scalp itching and flaking [14]. Therefore, it is necessary to improve and optimize the simple TDDS. The key to the functioning of the whole system is breaching the skin barrier.

**Figure 1:**
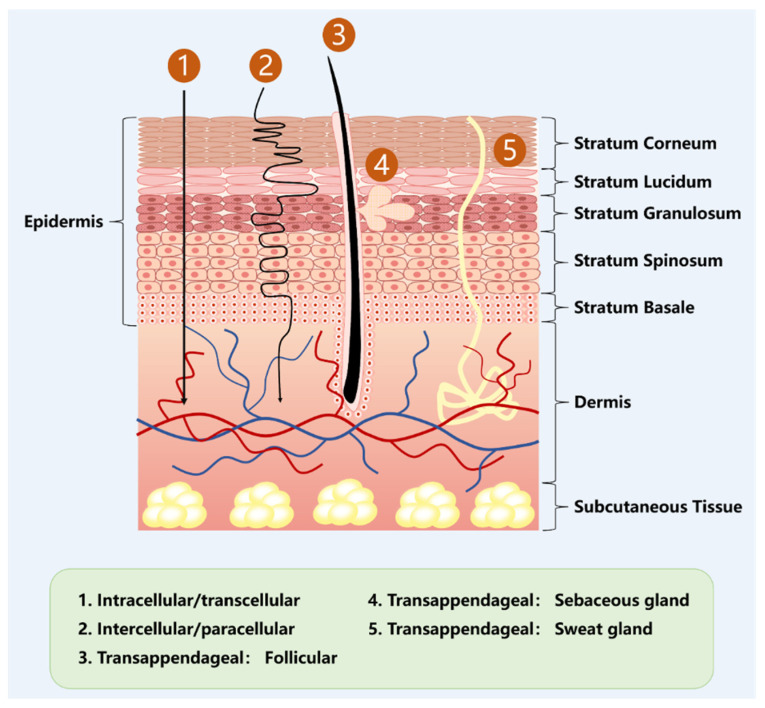
Anatomy of skin, along with skin permeation pathways, including transcellular, paracellular, and transappendageal. The figure was created with PowerPoint.

The skin is the largest organ in the human body, having a surface area of 2 m^2^ and a mass of 15% of total body weight, and it serves as a physical barrier to maintain homeostasis of the internal environment [15,16]. The skin is usually divided into three layers: epidermis, dermis, and subcutaneous tissue. The epidermis is further divided into the inactive epidermis (i.e., stratum corneum) and the active epidermis [17,18] (Figure 1). The stratum corneum is a significant barrier to drug penetration due to its unique arrangement of hydrophilic keratins and hydrophobic lamellar lipids in tightly packed layers [19]. Tightly packed adherent membrane proteins in the living epidermis also act as an essential resistance to drug penetration [20]. Active compounds can cross these barriers by specific mechanisms, such as transcellular (intracellular) penetration through SC keratinocytes, penetration through SC cell interstitial spaces, adnexal penetration through hair follicles, and penetration through sebaceous or sweat glands [21] (Figure 1). However, most drugs are limited by their molecular weight, lipophilicity, lipid–water partition coefficient, and other properties. Medical devices (e.g., microneedles) or nanocarriers must effectively cross the skin barrier and reach the focal site to exert therapeutic effects [22,23].

Therefore, this paper provides a detailed overview of recent studies on TDDS for the treatment of androgenetic alopecia. It covers the pathogenesis of AGA and the innovative application of functional drug-carrying nanoparticles. It contributes to developing a safer, more effective, and patient-friendly TDDS-based modality for treating AGA and lays the foundation for its translational innovation.

## 2. Pathogenesis and Common Treatments in Androgenetic Alopecia

### 2.1. Pathogenesis

#### 2.1.1. Androgen Metabolism

Androgens are particularly important in the pathology of AGA. 17β-Hydroxysteroid dehydrogenase and 3β-hydroxysteroid dehydrogenase/Δ5-4-isomerase are highly expressed in sebaceous gland cells, sweat glands, and dermal papilla cells. They can convert the adrenal production of relatively weak androgens, dehydroepiandrosterone sulfate, and androstenedione into more potent androgens such as testosterone. Testosterone is further irreversibly converted to dihydrotestosterone (DHT) by 5α-reductase, which binds specifically to the androgen receptor (AR) with strong affinity, and binding signals are transferred from the nuclear AR to the androgen response element in the promoter region, causing downstream gene regulation [24,25,26]. This ultimately leads to the miniaturization of hair follicles, gradually transforming the initially coarse terminal hair into fine and soft vellus [27].

#### 2.1.2. Immune Inflammation

Inflammation of the follicle microenvironment is also one of the triggers of AGA. It causes changes in the microenvironment of the hair follicle, which affects its normal growth and development, thus exacerbating the symptoms of hair loss. A study examined 347 tissue specimens collected from 23 patients with AGA and found that chronic inflammation marked by lymphocytes and histiocytes was present in about half of the tissue specimens [28]. Another research team studied 19 patients with AGA and 6 standard controls, taking occipital and cephalic biopsy specimens. The results also showed that the number of inflammatory infiltrates at the top of the head and the non-alopecia occipitalis was significantly higher in AGA patients than in controls [29].

### 2.2. Common Treatments

#### 2.2.1. Topical Minoxidil

Minoxidil is a highly effective vasodilator and potassium channel opener which was initially used for lowering blood pressure and later found to have the effect of promoting hair growth in clinical application. It can dilate blood vessels in the scalp, improve blood circulation around the hair follicles, provide more nutrients and oxygen to the hair follicles, and facilitate hair growth. It can also prolong the anagen period of hair follicles, shorten their resting period, and counteract the miniaturization of hair follicles [30]. Clinical trials have shown that topical 5% minoxidil solution can effectively promote hair growth [31,32]. However, it is worth noting that not all AGA patients respond clinically to minoxidil treatment. A clinical study showed that after 16 weeks of twice-daily application, less than 40% of patients responded, i.e., via hair regrowth [33]. Additionally, free minoxidil has low SC layer penetration, so it needs to be administered frequently, which can lead to side effects such as contact dermatitis, scalp itching, and peeling [14]. In addition, studies have shown that oral administration of 1 mg of minoxidil daily has the same therapeutic effect as topical application of 5% minoxidil solution [34,35]. However, side effects such as hirsutism, tachycardia, pericardial effusion, and lower extremity edema should not be ignored.

#### 2.2.2. Oral Finasteride

Finasteride is a 5α-reductase inhibitor, which converts testosterone to DHT, one of the key factors contributing to AGA. Finasteride reduces the production of DHT by inhibiting the activity of this enzyme, slowing down the process of hair follicle miniaturization and achieving the treatment of AGA [36]. Studies have shown that oral administration of 1 mg finasteride daily is more effective than topical application of 5% minoxidil [37]. However, the systemic effects of oral administration can lead to more serious sexual adverse effects, including erectile dysfunction, ejaculatory dysfunction, and gynecomastia [38]. They may also lead to an increased risk of depression [39].

#### 2.2.3. Hair Transplant

Two main types of hair transplantation procedures are available: follicular unit transplantation (FUT) and follicular unit extraction (FUE). In FUE, individual hair follicles are extracted and transplanted to the hair loss area of the scalp. Meanwhile, FUE offers additional advantages such as targeting groups of hair follicles of specific sizes and diameters, reduced postoperative pain and healing time, and increased hair follicles [40]. A retrospective study of 52 patients with AGA found significant improvement in hair density in patients younger than 33 after surgical treatment [41]. In another retrospective study of 1106 male AGA patients, the surgery was effective and significantly improved patient satisfaction with their appearance [42]. Additionally, it is important to note that the process from the start of the hair transplant surgery to the onset of its effects is a relatively lengthy one. Within 2–4 weeks post-surgery, the transplanted hair enters the telogen phase due to surgical stress, resulting in natural shedding. Between 3 and 6 months post-surgery, new hair begins to grow, with density gradually increasing. Initial results are visible by 6 months, but full maturation takes 12–18 months (with some patients requiring up to 24 months). During this period, careful scalp care is necessary to promote wound healing and prevent inflammation. The application of minoxidil must also be maintained for at least 5 weeks. If necessary, biotin or iron supplements may be taken orally in combination [6]. The entire process may cost between USD 4000 and USD 20,000. Therefore, although the final results of hair transplantation can be satisfactory, its obvious limitations cannot be ignored, including, for example, high invasiveness, long recovery period, complex postoperative care, and high cost [43].

In conclusion, although first-line treatments have been effective, they still have limitations such as side effects that are difficult to circumvent, high frequency of administration, high price, high invasiveness, and long recovery period, so there is an urgent need to develop new safe and effective treatments to provide more options for AGA patients.

## 3. Transdermal Drug Delivery (TDDS)

There are usually three routes for transdermal delivery of drugs: the trans-annexal route, the transcellular route, and the intercellular route, of which the trans-annexal route utilizes the hair follicle and its associated sweat glands for transdermal delivery. However, the hair follicle accounts for only 0.1% of the skin’s surface area; thus, this route is rarely used. Drugs mainly penetrate the skin through the transcellular and intercellular routes and diffuse into the dermis after passing through the stratum corneum [23]. Researchers have developed several novel transdermal drug delivery systems to break through the stratum corneum barrier and improve drug penetration. Their characteristics and specific applications are summarized in Table 1.

### 3.1. Microneedles (MNs)

Microneedles consist of needle-like structures ranging in length from 10 to 1000 µm [44], with specific mechanical properties. Therefore, they can rapidly penetrate the stratum corneum and form reversible microchannels to help the active drug reach the dermis, ultimately realizing transdermal drug delivery. At the same time, its micron-sized tip structure can ensure that it does not reach the capillary layer, so compared with the traditional subcutaneous injection drug delivery, MN administration is patient-friendly with minimal bleeding and low pain. Currently, five types of MNs, including solid MNs, coated MNs, hollow MNs (HMNs), dissolved MNs (DMNs), and hydrogel-forming MNs, have been used for percutaneous drug delivery [45,46], and their application in the field of treating AGAs has been well studied.

**Figure 2:**
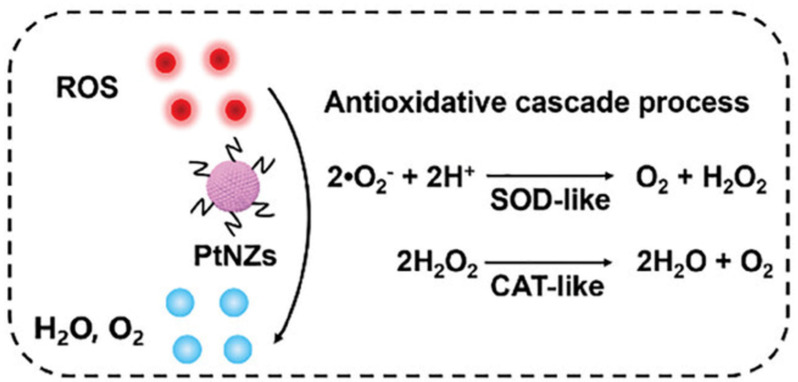
Schematic of SOD/CAT-like activity of PtNZs. Reprinted with permission from ref. [47]. Copyright 2024, with permission from John Wiley and Sons.

In 2024, Hu et al. designed a dissolving microneedle (Pt-MN) loaded with PtNZ. Platinum nano-enzymes (PtNZ) with superoxide dismutase (SOD) and catalase (CAT) mimetic activities can reduce reactive oxygen species (ROS) to oxygen in the pathological microenvironment of AGA (Figure 2), increase oxidative phosphorylation, and ultimately promote the differentiation of follicular stem cells for hair regeneration. However, PtNZ has poor skin permeability, whereas MN has sufficient mechanical strength to break through the skin barrier for effective deliver of PtNZ. Pt-MNs were prepared by simply loading PtNZs onto the tips of HA microneedles via centrifugation, combined with a PVP-K90 backing layer to provide mechanical support and controlled dissolution. Mechanical strength tests confirmed that the microneedles were capable of penetrating the skin, while confocal microscopy and energy-dispersive X-ray spectroscopy (EDS) revealed uniform loading of PtNZs within the microneedles. In vitro pig skin experiments showed that Pt-MNs could dissolve within 3 min and deliver drugs to the area around hair follicles at a depth of approximately 200 μm. Finally, this study achieved significant hair regeneration in an AGA model administered at a frequency of once every three days [47].

**Table 1:** Classification and Applications of TDDS.

TDDS	Characteristics	Applications		Reference
		Name	Components and Functions	
Microneedles (MNs)	Micron-sized needle-like structures can quickly penetrate the SC and form reversible microchannels to help active drugs reach the dermis.	Pt-MN	PtNZ: Eliminate ROS, improve the pathological microenvironment of AGA, and promote hair follicle stem cell differentiation. Dissolving MNs: Load PtNZ, penetrate the SC, dissolve, and release it.	[47]
		V-R-MN	Ritlecitinib: Inhibits JAK kinase activity, blocks excessive activation of the immune system that triggers attacks on hair follicles, and promotes hair regrowth. R-PHA NPs: Loaded with Ritlecitinib and combined with HA hydrogel to increase the mechanical properties of MNs. VEGF: Promotes blood vessel formation in hair follicles and provides nutrients for hair regeneration. Activates hair follicle stem cells and promotes hair growth by pushing hair follicles into the growth phase. HA-MN: Load and deliver the above functional ingredients.	[48]
Liposome	Liposomes are primarily composed of phospholipids and cholesterol. They form closed vesicles with a bilayer membrane structure through self-assembly in aqueous solutions, capable of encapsulating both hydrophilic and hydrophobic molecules simultaneously. They exhibit excellent biocompatibility, stability, deformability, and high drug-loading capacity.	HL@Mi/ NON Oate	Lecithin: Basic components of liposome membranes. Cho-PEI/ONOate: Cho forms a liposome membrane structure. PEI is a cationic polymer that regulates the surface charge of the carrier and adsorbs HA. Nitric oxide donors produce nitric oxide, which promotes vasodilation, enhances skin permeability, and increases the retention of minoxidil. At the same time, it stimulates angiogenesis, improves microcirculation in the hair follicles, and promotes the proliferation of dermal papilla cells. HA: Stabilize the lipid membrane structure. Improve the transdermal penetration efficiency of drugs. Minoxidil: By expanding the blood vessels in the scalp to increase the supply of nutrients and oxygen to the hair follicles, it promotes hair follicle cell proliferation and differentiation.	[49]
		CAR@Lip	Lipoid S75 and Cho: Membrane materials for liposomes, which encapsulate CAR to form a stable liposome structure. CAR: Regulating the expression of growth factors associated with the hair follicle cycle, such as IGF-1, VEGF, KGF, and TGF-β; participating in the SHH/Gli and Wnt/β-catenin signaling pathways, thereby promoting the transition of hair follicles from the telogen phase to the anagen phase and promoting hair regrowth. However, CAR has poor water solubility and low bioavailability, limiting its application in transdermal delivery. Therefore, it is encapsulated in liposomes.	[50]
Ionic liquid (IL)	Ionic liquids are composed of organic cations and organic/inorganic anions. On the one hand, anions and cations can interact with keratin layer lipids through electrostatic forces or hydrogen bonds, disrupting the ordered structure of lipids and enhancing their fluidity. On the other hand, ionic liquids can both increase the solubility of hydrophobic drugs and reduce the charge barrier of drugs by forming neutral ion pairs. These properties enable ionic liquids to combine the high efficiency of traditional permeation enhancers with excellent biocompatibility.	GHK-Cu/ CaT-ME	GHK-Cu: Increases VEGF expression. Promotes new blood vessel formation, providing nutrients for hair growth; promotes hair papilla cell proliferation and inhibits apoptosis; inhibits TGF-β production, preventing hair follicles from prematurely transitioning from the growth phase to the resting phase. CaT: An ionic liquid composed of tartaric acid and L-carnitine, which has hair growth properties. It can also act as a polar phase to form a microemulsion with IPM, improving the local delivery efficiency and bioavailability of copper peptides. IPM: As a nonpolar phase, it forms microemulsions with CaT and surfactants.	[51]
		ME@CGIL	CGIL: Choline and geranic acid are synthesized into IL through a double decomposition reaction in a single step. This process enhances the solubility of MXD and EGCG, improves their transdermal penetration, and prolongs their retention time in the skin. Additionally, CGIL itself possesses certain hair growth-promoting functions, stimulating angiogenesis around hair follicles. EGCG: A natural plant extract with excellent antioxidant activity that effectively reduces ROS levels and inhibits ROS-induced cell apoptosis, regulates oxidative stress in the hair follicle microenvironment. Minoxidil: By expanding the blood vessels in the scalp to increase the supply of nutrients and oxygen to the hair follicles, it promotes hair follicle cell proliferation and differentiation.	[52]
Nanostructured lipid carrier (NLC)	Nano-structured lipid carriers (NLC) are composed of an aqueous phase, a solid-liquid mixed lipid phase, and surfactants acting in synergy. When applied to the skin surface, the water in the NLC evaporates, and the lipid components spontaneously arrange themselves through intermolecular forces, forming a dense and continuous film. This reduces water loss, improves skin hydration, and promotes drug penetration. The lipid components can also disrupt the lipid arrangement in the stratum corneum, temporarily breaking down its tight structure to form microchannels, thereby enhancing drug diffusion.	DST-NLC	Stearic acid: Forming a lipid matrix, it is one of the main lipid components of NLC. Phosal^®^ 53 MCT: Together with stearic acid, it forms a stable lipid matrix structure. Lutrol^®^ micro68: Surfactants reduce interfacial tension. CSO-LA: Synthesized from chitosan oligomers and lauric acid, it is used to coat DST-NLC. Chitosan oligomers carry a positive charge due to their amino groups, promoting the adsorption and penetration of the carrier in the skin and hair follicle areas. Lauric acid has strong anti-androgenic activity, inhibiting 5α-reductase activity, and synergistically with dutasteride to prevent hair loss and promote hair growth. Dutasteride: A dual type I and type II 5α-reductase inhibitor that reduces serum DHT levels.	[53]
		SL-NLC	Compritol^®^888 ATO: As a solid lipid, it forms NLC. It can also dissolve lipophilic drugs, helping to increase the loading capacity of SL. Olive oil: A liquid lipid in NLC that can interfere with the crystal order of solid lipids during preparation, increasing the space available for drug molecules. Transcutol^®^P: Added to NLCs as a penetration enhancer, it increases drug penetration into the skin. It also reduces the surface tension of oil droplets, promoting the formation of a uniform emulsion and the preparation of nanoparticles with uniform particle size. SL: Has powerful anti-androgenic properties, acting by reducing androgen production and competitively blocking androgen receptors in target tissues.	[54]
Transformers (TFs)	Its structure is similar to that of liposomes, but edge active agents are embedded in the lipid bilayer. It can endow TFs with additional flexibility, enabling them to deform under the drive of the osmotic pressure gradient of the skin, overcome the size limitations of traditional nanoparticles, and penetrate pores in the stratum corneum that are much smaller in diameter, thereby achieving targeted drug delivery to the deep layers of the skin.	MXD-Rg3@TFs	Rg3: By targeting the PI3K/AKT and MAPK/NF-κB signaling pathways and inflammatory factors, it potentially regulates inflammation and oxidative stress-related pathways. Its steroid structure is similar to cholesterol, and it can replace cholesterol in the preparation of TF, thereby avoiding the disruption of steroid homeostasis or steroid-sensitive signaling pathways in the body that may be caused by cholesterol accumulation in hair follicles. Minoxidil dilates blood vessels, increases blood flow to hair follicles, stimulates dermal cells to produce growth factors, and activates the β-catenin pathway, thereby inducing and prolonging the growth phase of hair and promoting hair growth.	[55]

**Figure 3:**
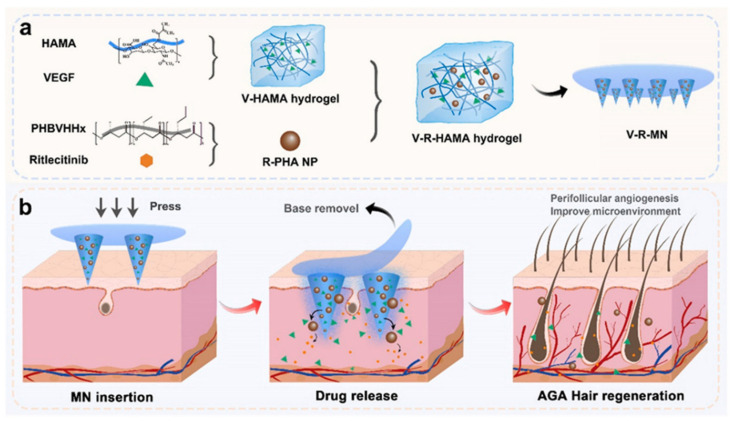
Schematic illustration of the V-R-MN integrated microneedle patch for androgenetic alopecia treatment. (**a**) Fabrication of V-R-MN. (**b**) The application of the V-R-MN patch induces appropriate mechanical stimulation, and the VEGF released by V-R-MN in the skin remodels the microvascular network around the hair follicle, and the Ritlecitinib released by the V-R-MN improves the immune microenvironment around the hair follicle, thus promoting hair follicle development and hair regrowth. Reprinted with permission from ref. [48]. Copyright 2024, with permission from Elsevier.

In the same year, Ding et al. addressed the downregulation of angiogenic genes and insufficient vascularization in the hair follicle microenvironment of AGA patients. They designed a functional MN that integrates the promotion of angiogenesis, improvement of the hair follicle microenvironment, and promotion of follicle cell proliferation and development. The design used slowly degradable polyhydroxy fatty acid ester (PHA) nanoparticles (R-PHA NPs) encapsulated with the novel hair loss drug Ritlecitinib, co-loaded with vascular endothelial growth factor (VEGF) in hyaluronic acid (HA) hydrogel microneedle (V-R-MN) (Figure 3). Among them, the cumulative release rate of VEGF over 28 days exceeded 80%; the release rate of Ritlecitinib over 28 days was approximately 69%. PHA NPs were prepared using an emulsification–solvent evaporation method and loaded with Ritlecitinib. SEM analysis revealed that the NPs were spherical and uniformly dispersed, with particle sizes uniformly distributed between 150 and 200 nm. MNs were formed using a PDMS mold via UV crosslinking. SEM observation showed that the needle tips were structurally intact, with a height of approximately 500 μm. The combination of HA hydrogel and PHA nanoparticles significantly enhances the mechanical properties of MNs. Mechanical testing indicated that the addition of R-PHA NPs significantly enhanced the fracture resistance of micro-needles, with a fracture rate of only 3.5% under a 200 g load, which help break through the keratin barrier while stimulating angiogenesis. In vitro experiments showed that fluorescently labeled drugs can penetrate the stratum corneum of pig skin via microneedles and accumulate in the epidermis and dermis layers at a depth of 200–300 μm around the hair follicles. In vivo experiments showed that after administration to AGA model mice, the drug primarily distributed in the skin at the treatment site, co-localized with the hair follicle cycle activation marker Ki67, and was largely degraded within 11 days. No significant residues were observed in skin tissue sections, and no fluorescent signals were detected in major organs, confirming its safety and local action characteristics. In AGA model mice, V-R-MN resulted in rapid onset of hair growth over time, improved quality, and greater coverage, with significantly more potent efficacy than single drug minoxidil [48].

### 3.2. Liposome

Liposomes can be formed from a combination of selected phospholipids and cholesterol which can self-assemble in an aqueous environment to form enclosed vesicles containing the aqueous fluid bounded by one or more (concentric) bilayer membrane structures [56]. Their core feature is the ability to encapsulate both hydrophilic and hydrophobic molecules. Hydrophilic substances can be encapsulated in the internal aqueous phase core, while hydrophobic components can be embedded in the hydrophobic region of the phospholipid bilayer. Liposomes have good biocompatibility, stability, deformability, and high drug-carrying capacity [57]. Drugs encapsulated by liposomes can enhance the efficiency of transdermal absorption and effectively reduce the toxic side effects caused by local and systemic drug absorption. It is an ideal carrier for transdermal delivery [58]. Liposomes show crucial therapeutic potential in skin therapy as a carrier for topical drug delivery [59]. They have been applied in treating various skin diseases, such as alopecia, acne, scars, psoriasis, vitiligo, and melanoma [60,61].

**Figure 4:**
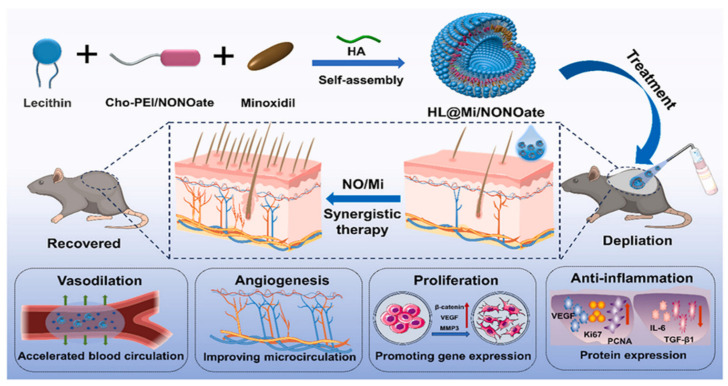
Schematic diagram of HL@Mi/NONOate transdermal delivery platform design route and synergistic treatment of androgenetic alopecia. Reprinted with permission from ref. [49]. Copyright 2024, with permission from Elsevier.

To address the problems of ineffectiveness and side effects of free minoxidil in the treatment of hair loss, Xing et al. constructed a novel drug delivery system by loading minoxidil onto hyaluronic acid liposomes (HL@Mi/NON Oate) [49] (Figure 4). The system utilizes three mechanisms to synergize the therapeutic effect. Nitric oxide (NO) promotes capillary dilation and accelerates blood flow, facilitating minoxidil penetration. The advantage of a slow-release drug in liposomes allows minoxidil to exert a long-lasting effect in the skin. Binding of minoxidil to the signaling molecule NO promotes cell proliferation and angiogenesis, downregulates the expression of inflammatory factors, and inhibits the inflammatory response of hair follicles. At the same time, it upregulates the expression of Ki-67 and PCNA proteins in hair follicle tissues, induces hair follicle regeneration and development, and ultimately realizes multi-pathway synergistic treatment of AGA. The lipid-based composite delivery system was synthesized using the reverse evaporation method. First, cholesterol chloroformate was used to modify polyethyleneimine to prepare the NO donor Cho-PEI/NONOate. This was then self-assembled with soybean lecithin, hyaluronic acid, and minoxidil to form liposomes. The structure was confirmed via ^1^H NMR, FTIR, UV-Vis, and other characterization techniques to confirm the structure. Fluorescence labeling showed that hyaluronic acid was uniformly distributed in the interlayer space of the liposomes, and TEM observation revealed a uniform morphology. The hydrated particle size was less than 500 nm, and after drying, the particle size stabilized at approximately 200 nm, exhibiting a spherical closed vesicle structure with a Zeta potential of −24 mV and good stability. The minoxidil loading capacity was approximately 7% lower than that of the control group HL@Mi, but still met therapeutic requirements. The NO loading capacity was 1.75 μmol/mg, exhibiting a “fast-then-slow” sustained-release profile at pH 7.4, with approximately 60% released within 5 h and nearly complete release by 36 h. Pharmacokinetic studies of this system indicate that NO dilates capillaries by activating the cGMP pathway, resulting in skin blood flow on the mouse back reaching 4.2 times that of the control group within 5 min post-administration, significantly enhancing minoxidil transdermal penetration efficiency. The skin retention levels at 2 h were significantly higher than those of commercially available solutions and the HL@Mi group. Fluorescently labeled Rhodamine B indicated that HL@Mi/NONOate penetrated deeper, with higher fluorescence intensity in the dermis and muscle tissues.

Adverse effects such as contact dermatitis and facial hirsutism are known to be associated with topical administration of minoxidil [62,63]. Oral finasteride causes sexual dysfunction and mental and psychological disorders [64]. Therefore, natural plants that can treat AGA with fewer side effects need to be developed. Cardamonin (CAR) is a flavonoid that can significantly improve hair growth. However, it has poor water solubility and low bioavailability. Therefore, Liu et al. developed a CAR-loaded liposome formulation (CAR@ Lip) to significantly improve the solubility of CAR. CAR@Lip was prepared via thin-film dispersion and ultrasonication, resulting in uniformly spherical particles with an average particle size of 137.03 ± 1.56 nm, a drug loading capacity of 6.09%, and an encapsulation efficiency of 96.56%. In vitro release studies showed that both CAR@Lip and the gel significantly enhanced the cumulative drug release rate, with faster release in the later stages. Transdermal experiments indicated that both formulations significantly enhanced CAR transdermal permeability, particularly in hair follicles, where drug retention was 68.79 times and 10.75 times that of free drug, respectively. In vivo experiments confirmed that both formulations promote hair growth in AGA model mice and accelerate the transition of hair follicles to the anagen phase, with mechanisms related to regulating the expression of IGF-1, VEGF, KGF, TGF-β, and activating the SHH/Gli and Wnt/β-catenin signaling pathways, and no significant skin irritation, demonstrating potential for treating AGA [50].

**Figure 5:**
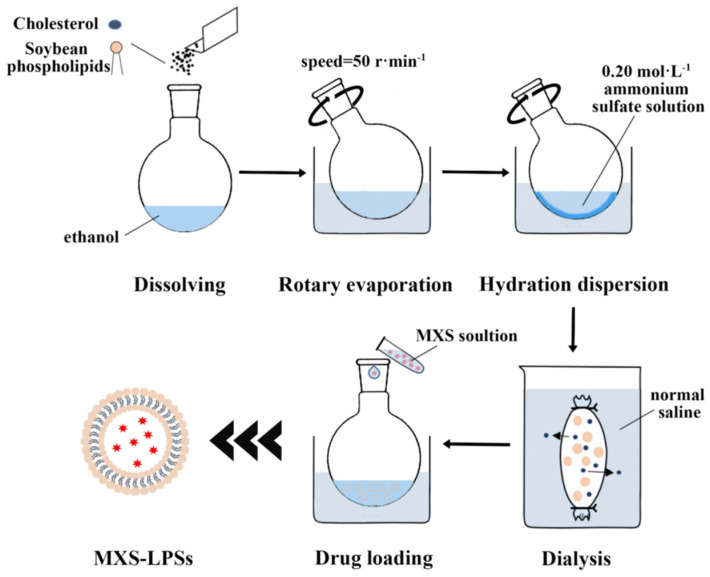
The optimal formulation and manufacturing process of MXS-LPSs. Reprinted with permission from ref. [65]. Copyright 2025, with permission from Elsevier.

Minoxidil sulfate (MXS) is a weak base strong acid ester, which is a phase II active metabolite generated by the action of sulfotransferase in hair follicles on minoxidil. MXS acts as a potassium channel opener, and its mechanism of stimulating hair growth is similar to that of MX, but its in vitro activity is 14 times higher than that of minoxidil. However, its poor stability in aqueous solution makes it difficult to deposit at the hair follicle efficiently, and it has low bioavailability. Therefore, Shan et al. prepared a novel formulation of MXS-LPSs by a combination of thin-film hydration-dispersion method and ammonium sulfate gradient active drug loading process, resulting in a semi-transparent, blue-tinged emulsion. Under transmission electron microscopy, they appear as uniformly spherical particles (Figure 5). The average particle size is 129.46 ± 7.04 nm, the zeta potential is −25.57 ± 4.79 mV, the encapsulation efficiency reaches 92.72 ± 0.75%, and the drug loading capacity is 2.80 ± 0.12%. In vitro transdermal experiments showed that the cumulative transdermal amount of MXS-LPSs at 36 h was 225.98 ± 11.53 μg·cm^−2^, which was lower than that of the MXS aqueous solution, but the proportion of drug deposition in the skin beneath the stratum corneum reached 59.80 ± 26.27%, significantly higher than that of the MXS solution (32.55 ± 1.61%) and minoxidil solution (43.18 ± 6.58%). Laser confocal microscopy confirmed that MXS-LPSs accumulated significantly in the hair follicle region through particle size matching (<300 nm), negative charge adsorption, and the affinity between phospholipids and sebum, with fluorescence intensity 2.27 times that of the water-soluble control Rho-B. In a rat model of androgenetic alopecia, after 45 days of treatment, the MXS-LPSs group showed significantly superior hair length, weight, and growth scores compared to the minoxidil solution group. Hair follicles exhibited a marked transition from the telogen to anagen phase, with no skin irritation or sensitization reactions. This demonstrates the advantages of targeted hair follicle delivery, enhanced efficacy, and safety [65].

### 3.3. Ionic Liquid (IL)

Ionic liquids are room temperature molten salts composed of organic cations and organic/inorganic anions [66,67,68]. Studies have shown that ILs synergistically promote drug transdermal absorption through multiple mechanisms: on the one hand, their anions and cations can interact with stratum corneum lipids (e.g., ceramides, cholesterol) by electrostatic or hydrogen bonding to disrupt the lipid ordering structure and enhance mobility; on the other hand, ILs can both enhance hydrophobic drug solubility and reduce the charge barrier of the drug through the formation of a neutral ion pair. In addition, ILs can reversibly alter stratum corneum protein conformation, temporarily expanding intercellular channels while maintaining skin integrity. These properties enable ILs to combine the high efficiency of traditional osmotic enhancers with superior biocompatibility and show promising applications in TDDS [69,70,71].

**Figure 6:**
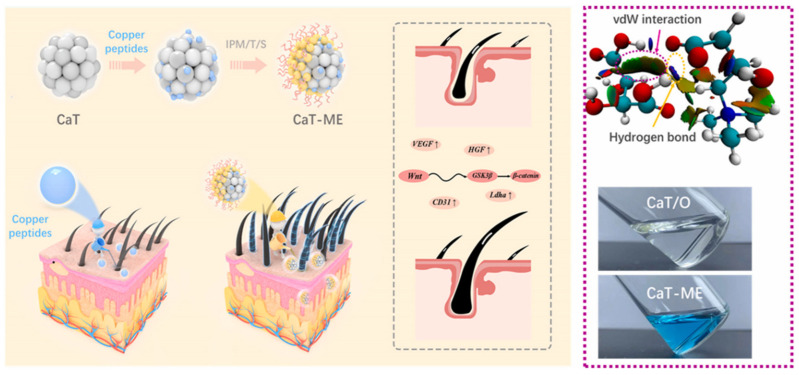
Schematic diagram of GHK-Cu/CaT-ME transdermal delivery platform design route and synergistic treatment of androgenetic alopecia. Reprinted with permission from ref. [51]. Copyright 2024, with permission from Elsevier.

Copper peptide (GHK-Cu) is a bioactive complex formed by chelation of glycine-histidine–lysine tripeptide with copper ions (Cu^2+^), which has a powerful ability to promote hair growth. The principle of action is the following: (i) stimulate fibroblasts to produce VEGF, promote the generation of new blood vessels, and provide the required nutrients for hair growth [72,73,74]; (ii) promote the proliferation of hair papilla cells and inhibit their apoptosis, stimulating the growth of hair follicles and hair [75,76]; (iii) inhibit the production of TGF-β to prevent hair follicles from prematurely changing from anagen to degeneration [77,78,79,80]. However, the copper element in GHK-Cu is in a sub-stable valence state, which is extremely sensitive to pH, antioxidants, chelating agents, and other factors, and has poor stability [81,82]. Moreover, GHK-Cu is highly hydrophilic, and when applied topically to the skin, the amount absorbed is extremely low [83]. If injected into the dermis, about 95% of the peptide is metabolized and excreted from the body, with extremely low bioavailability [84]. Therefore, Liu et al. designed a thermodynamically stable ionic liquid microemulsion (IL-M) through theoretical calculations and pseudo-ternary phase design. The system uses ion liquid (CaT) synthesized from naturally sourced L-carnitine and tartaric acid via an amidation reaction as the polar phase, combined with isopropyl myristate (IPM) as the non-polar phase, and Tween 80 and Span 20 as surfactants to form a water-in-oil (W/O) microemulsion (Figure 6). Characterization revealed that the average particle size of CaT-ME was 55.21 ± 1.62 nm, with a uniform spherical shape. The solubility of copper peptides in CaT exceeded 130.98 g/L, and the structure remained stable after loading. In vitro transdermal experiments showed that CaT-ME increased the cumulative penetration of copper peptides in pig skin to 3.18 times that of the PBS group, with fluorescence intensity 3.9 times higher, demonstrating the ability to penetrate the stratum corneum and accumulate in the dermis and subcutaneous tissue. In vivo experiments in mice showed that hair follicles in the CaT-ME group entered the anagen phase within 6 days, and hair density was significantly higher than that in the minoxidil group after 28 days. The mechanism is associated with the activation of the Wnt/β-catenin signaling pathway, promotion of vascular endothelial growth factor (VEGF) expression, and activation of hair follicle stem cells. This system combines biocompatibility and stability, providing an efficient local delivery strategy for non-invasive treatment of hair loss [51].

**Figure 7:**
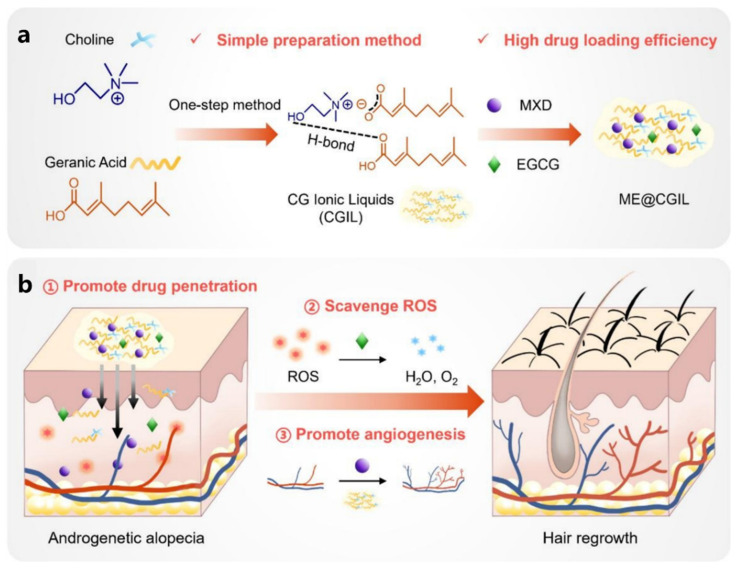
Schematic diagram illustrating the mechanism of action of ME@CGIL for AGA treatment. (**a**) The synthetic route of ME@CGIL, where CGIL is formed through double decomposition reaction between choline and geranic acid. ME@CGIL refers to CGIL loaded with MXD and EGCG. (**b**) The basic mechanism of ME@CGIL for AGA therapy: Upon topical application, EGCG enters the epidermis and dermis to remove excess ROS from the microenvironment, while CGIL and MXD could induce perifollicular angiogenesis. Subsequently, hair follicles transform from the telogen phase to the anagen phase, promoting hair regrowth. Reprinted with permission from ref. [52]. Copyright 2024, with permission from Elsevier.

It is known that minoxidil stimulates the proliferation and differentiation of hair follicle epithelial cells through the modulation of vascularization, resulting in the reactivation of atrophied hair follicles, promoting hair regrowth [85]. However, it is often difficult to achieve the desired effect with minoxidil alone [4]. Epigallocatechin gallate (EGCG) is a natural plant extract. It has excellent antioxidant activity and can effectively reduce ROS levels and inhibit ROS-induced apoptosis. The synergistic effect of the two can realize the effective treatment of AGA, but its low solubility and low permeability are the key to the drug delivery problem. Therefore, Luo et al. constructed a novel multifunctional IL carrier (CGIL) based on choline and geranylgeranylate, which significantly improved the solubility of minoxidil and realized the efficient co-loading of two active ingredients, minoxidil and EGCG (ME@CGIL) [52] (Figure 7a). The carrier CGIL is synthesized from choline and gallic acid via a one-step metathesis reaction, resulting in a pale yellow transparent liquid. Its structure was confirmed via NMR and FT-IR spectroscopy. It dissolves drugs through hydrogen bonding to form a homogeneous solution, with a drug loading capacity of 50 mg/mL for minoxidil and 10 mg/mL for EGCG. In vitro transdermal experiments showed that CGIL significantly enhances drug penetration, with cumulative penetration levels of 275.4 μg/cm^2^ for minoxidil and 34.3 μg/cm^2^ for EGCG, with skin retention levels of 3427.0 μg/g and 824.2 μg/g, respectively, and the drugs remained in the skin for up to 72–96 h. In an androgenetic alopecia mouse model, ME@CGIL promoted angiogenesis around hair follicles and activated hair follicle stem cells by clearing excess ROS, MXD, and CGIL through EGCG, resulting in a hair regrowth coverage rate of 84.98% (Figure 7b), significantly outperforming commercially available minoxidil (47.41%), with lower dosage and frequency and superior quality of regrown hair. This system is simple to prepare and has good biocompatibility, offering an efficient and low-toxicity novel therapeutic strategy for androgenetic alopecia.

### 3.4. Nanostructured Lipid Carrier (NLC)

Nanostructured lipid carrier (NLC) is a composite system consisting of an aqueous phase, a lipid phase mixed with solid–liquid, and a surfactant synergistically. During self-assembly, liquid lipids are embedded in solid lipid lattices, forming imperfect crystal structures (defect states) and creating porous or disordered lipid networks at the nanoscale. This structure increases drug encapsulation efficiency and controls release rates [86]. After application to the skin surface, the water in the NLC evaporates, and the lipid components spontaneously align through intermolecular forces (e.g., van der Waals forces, hydrophobic interactions) to form a dense and continuous film. It reduces water loss, improves skin hydration status, and promotes drug penetration [87,88]. The lipid components in NLC can also disturb the lipid arrangement of the stratum corneum, temporarily destroying the tight structure of the stratum corneum, forming microchannels, and further promoting drug diffusion. At the same time, NLC has a high drug encapsulation rate, which can effectively improve drug stability and ultimately realize the improvement of drug bioavailability and therapeutic effect [89]. In addition, by its nanoscale size regulation and surface charge modification, NLC can also precisely target specific tissues or cells [90]. Based on the above advantages, NLC has become a promising carrier system for local transdermal drug delivery and has shown a broad application prospect in the field of drug delivery.

Dutasteride is a type I and type II dual 5α-reductase inhibitor, biologically more potent than finasteride. It reduces serum DHT by more than 90% [91]. However, because of this, patients receiving oral dutasteride have a greater likelihood of experiencing decreased libido, increased depression, and ejaculatory disorders [92]. Therefore, Norhayati Mohamed Noor et al. [53] synthesized lauric acid–chitosan oligomer (CSO-LA) using lauric acid as a raw material, encapsulated with dutasteride to make a nanostructured lipid carrier (DST-NLC) for topical delivery to minimize the side effects associated with systemic circulation. In addition, lauric acid itself has anti-androgenic and pro-proliferative activities in hair follicle dermal papilla cells, which can synergize with dutasteride. CSO-LA was prepared using the melt dispersion–ultrasonication method and synthesized via EDC·HCl coupling, then characterized by FTIR, ^1^H NMR, and ninhydrin assay. Uncoated NLCs had a particle size of approximately 184 nm and a ζ potential of −18.0 mV. After coating, the particle size slightly increased to 188 nm, the ζ potential shifted to +24.8 mV, and the PDI remained <0.3. The physical stability was maintained for 180 days, with an encapsulation efficiency of 97% and a drug loading capacity of 3.49%. In vitro release studies showed that the CSO-LA-coated group released 55% of the drug within 24 h, slower than the 80% release of the uncoated group, and neither group exhibited drug penetration through pig ear skin, with the drug primarily retained in the epidermis and hair follicles. Cytotoxicity tests showed that the IC_50_ values for both coated and uncoated DST-NLCs were higher than those for free drug, and at 25 µM, they promoted cell proliferation. EpiDerm™ testing revealed no skin irritation. Fluorescent labeling indicated that the skin and cellular uptake of both coated and uncoated NLCs exhibited time-dependent patterns. This system enhances skin affinity through the positive charge of CSO-LA, enabling local sustained release, reducing systemic exposure and toxicity, and holds promise for the treatment of AGA.

Spironolactone (SL) has potent antiandrogenic properties and can be used for the oral treatment of AGA. There are two mechanisms of action: reduction in androgen production and competitive blockade of androgen receptors in target tissues [54]. SL produces efficacy after oral administration as demonstrated in many case reports and trials [83,93], but the regimen usually produces dose-dependent adverse effects. Therefore, Rehab Shamma et al. prepared colloidal NLC loaded with SL for topical delivery by emulsification solvent diffusion and evaporation. The prepared NLCs were spherical, with particle sizes ranging from nanoscale (215.6–834.3 nm) and a drug encapsulation rate of 74%. The drug release behavior of SL in NLCs was characterized by a pre-sudden release and a late, slow release. Confocal laser scanning microscopy confirmed the ability of SL-NCL to target and enrich at hair follicles [94].

### 3.5. Transfersomes (TFs)

Transfersomes (TFs), as a revolutionary member of the liposome family, have redefined the boundaries of transdermal delivery through their unique flexible vesicle structure. Their core components are phosphatidylcholine and cholesterol, based on which the edge-active agent is innovatively embedded in the lipid bilayer [95]. Similar to standard liposomes, the nanoscale size of TF allows it to pass through the interstices of the lipid matrix of the stratum corneum. The edge-active agent, on the other hand, gives it additional flexibility. Edge-active agents are typically single-chain surfactants (such as bile salts or Tween 80). Their primary function is to enhance the flexibility and deformability of the lipid bilayer, enabling the carrier to pass through skin keratin layer pores that are significantly smaller than its own size. The mechanism involves: inserting into the phospholipid bilayer via the amphiphilic structure within the molecule, disrupting the compact arrangement of lipids, reducing membrane rigidity and interfacial tension; simultaneously responding to the osmotic pressure generated by the skin’s hydration gradient to drive active deformation and directed penetration of the vesicle, overcoming the size limitations of traditional nanoparticles, and traversing pores in the stratum corneum that are significantly smaller than their diameter, thereby achieving targeted drug delivery to deeper layers of the skin. The synergistic effect of this highly elastic structure and osmotic gradient significantly improves active pharmaceutical ingredients’ capture efficiency. It realizes controlled slow release in dermal target sites such as hair follicles [96]. Because of this, TF, as a carrier-loaded active drug delivery system, provides a superior option for AGA therapy [97].

In study [98], different lipid bilayer nanoparticles were evaluated for their hair follicle targeting capability in the delivery of dutasteride. The researchers evaluated their hair follicle targeting ability using dutasteride as a model drug, including conventional liposomes (LPPC), cholesterol-containing rigid liposomes (LP Chol), and edge-activator-containing flexible liposome transporters (TFs). These lipid nanoparticles were characterized by particle size, zeta potential, and encapsulation rate using oily dutasteride solution as a control. In vitro drug release, skin penetration assays, and hair follicle targeting factor calculation were performed. The results showed that TF had a cohesive core and smaller particle size, and the encapsulation rate was 88.1%. In the in vitro drug release experiment, the release curve of TF was close to that of the oily solution, and the release at 12 h was 13.5 ± 1.5%, which was a reasonable control of the release effect. In the skin penetration experiment, LP Chol promoted the accumulation of dutasteride in the hair follicle at 12 h. Still, the targeting effect of TF on the hair follicle was more significant, with a targeting factor of 0.32, which was significantly better than that of other liposomes. Regarding the body, TF has better control of dutasteride release and better follicle targeting in the preperiod, which is a more suitable choice for topical dutasteride delivery. However, the formulation still needs to be optimized, and future clinical studies are required to evaluate the safety of long-term use. This provides an essential reference for studying transdermal treatment of AGA.

**Figure 8:**
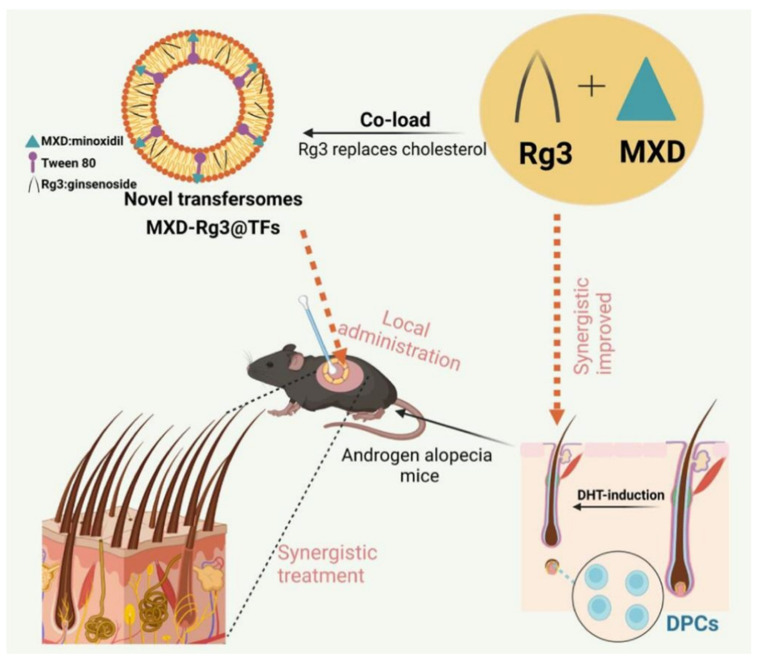
Schematic diagram of MXD-Rg3@TFs transdermal delivery platform design route and synergistic treatment of androgenetic alopecia. Reprinted with permission from ref. [55]. Copyright 2024, with permission from Elsevier.

In TF preparation, cholesterol improves the stability and fluidity of phospholipid bilayers and is a conventional material for forming lipid membranes [99]. However, cholesterol is a precursor for steroid hormone synthesis used to biosynthesize testosterone. Ginsenoside Rg 3 (Rg3) is a pharmacodynamic constituent of ginseng medicinal plant extracts with a steroidal structure similar to cholesterol [100]. Rg3 can potentially moderate inflammation and oxidative stress-related pathways by targeting the PI3K/AKT and MAPK/NF-κB signaling pathways and inflammatory factors [100]. Thus, the preparation of TF using Rg3 as a cholesterol substitute can avoid potential disruption of sterol homeostasis or sterol-sensitive signaling pathways in vivo due to cholesterol accumulation in hair follicles [101,102], as well as scavenging of intracellular ROS and reduction of inflammation levels. Therefore, Liu et al. prepared novel TFs with Rg3-substituted cholesterol material and loaded with minoxidil (MXD-Rg3@TFs) (Figure 8). The transfer body was synthesized using the thin-film hydration method, with soybean lecithin as the lipid material, Rg3 replacing cholesterol, and Tween 80 as the edge activator. It has a particle size of approximately 84 nm, a uniform spherical shape, and deformation capabilities similar to those of cholesterol-containing transfer bodies (MXD-Ch@TFs), but with superior stability and an encapsulation rate of 89.51%. In vitro experiments demonstrated that the combination of MXD and Rg3 significantly promoted DHT-induced proliferation of DPCs, reduced intracellular ROS and inflammatory factor levels, and inhibited cellular senescence. In vivo experiments showed that in the C57BL/6 mouse AGA model, MXD-Rg3@TFs were more effective than MXD-Ch@TFs and commercially available tinctures in shortening the hair follicle telogen phase, prolonging the anagen phase, and increasing hair length and thickness. This effect is related to the inhibition of testosterone conversion to DHT, reduction in inflammatory factors (IL-6, TNF-α) levels, and it is non-irritating to the skin. It holds promise for enhancing AGA treatment efficacy through multi-mechanism synergy while reducing drug dosage [55].

## 4. Conclusions and Perspective

**Figure 9:**
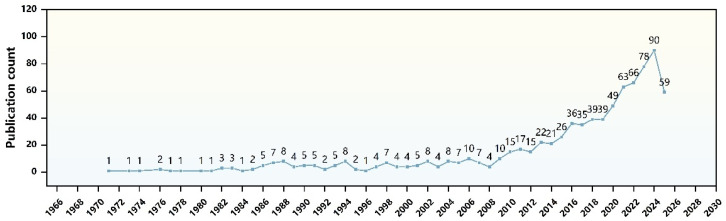
The annual publication trends of thec literature retrieved from Scopus using the keywords “androgenetic alopecia” and “transdermal drug delivery” are presented. The figure was created with Origin.

We conducted a comprehensive search in the Scopus database using keywords such as “androgenetic alopecia” and “transdermal delivery”, with the annual publication output over the past six decades illustrated in Figure 9. Through systematic review, critical analysis, and synthesis of the identified literature, we compiled and presented this review article, which systematically summarizes the innovative application of TDDS in AGA treatment, revealing its core mechanism of breaking through the skin barrier (e.g., stratum corneum and hair follicle microenvironment). Through the integration of microneedles, liposomes, ionic liquids, nanostructured lipid carriers, and transformers, TDDS significantly enhances the local penetration efficiency and bioavailability of drugs. For example, microneedles deliver platinum nano enzymes or pro-angiogenic factors through physical penetration to activate hair follicle stem cell differentiation. Liposomes and TFs enable slow release and targeted delivery of drugs such as minoxidil and dutasteride, reducing the risk of systemic side effects. IL-based microemulsion systems and Rg3-substituted cholesterol TFs (MXD-Rg3@TFs) have demonstrated superior efficacy and safety by enhancing drug stability and anti-inflammatory effects. These technologies not only overcome the limitations of conventional therapies (e.g., sexual dysfunction with finasteride, skin irritation with minoxidil, and high cost of hair transplantation) but also enable precision therapy through synergistic multi-mechanisms (e.g., anti-androgenic, antioxidant, and pro-angiogenic).

However, despite breakthroughs in overcoming skin barriers and improving local delivery efficiency, the clinical translation of novel carrier systems remains constrained by three major barriers: the gap between technical idealization and practicality, the superficiality of mechanism studies, and the systematic neglect of commercial viability. The high efficiency of carriers such as microneedles and liposomes in animal models (e.g., Pt-MN promotes hair regrowth with once-every-three-days administration) masks the lack of human validation. Human skin thickness, hair follicle density, and immune microenvironment differ significantly from those of mice, yet most studies rely on mouse back models, ignoring critical factors such as the thicker scalp stratum corneum and heterogeneous hair follicle distribution. More paradoxically, the complexity of carriers’ conflicts with scalable production: for example, ionic liquid microemulsions (GHK-Cu/CaT-ME) require precise pseudo-ternary phase design, and some liposomes have a drug loading rate of only 7% (HL@Mi/NONOate), raising questions about industrialization costs and stability. Regarding mechanisms, studies generally attribute efficacy to “activating the Wnt pathway” or “clearing ROS,” but lack dynamic analysis of target interactions. For example, Rg3-substituted cholesterol carriers (MXD-Rg3@TFs) claim to exert anti-inflammatory effects via the PI3K/AKT pathway, but the weight of this pathway in AGA hair follicle cells has not been validated, nor has the difference in signal disruption compared to traditional cholesterol carriers been compared. This “mechanism packaging” leads to technological homogenization—all five carriers claim “anti-androgen + pro-angiogenic” effects, yet no data reveal which carrier truly optimizes follicular pharmacokinetics. In terms of safety and commercial viability, the skin accumulation risk of cationic nanocarriers (e.g., CSO-LA-NLC), the allergenicity of lipid components (soybean phospholipids), and the disruption of skin microbiota by ionic liquids have not been assessed. The high cost of hair transplantation surgery is a given, and the economic viability of the carriers themselves cannot be ignored. For example, the single-dose cost of platinum nanocatalyst microneedles (Pt-MN) or recombinant protein carriers (VEGF-R-MN) can be equally high, and their cost-effectiveness remains questionable.

Therefore, the value of TDDS lies not in the complexity of the carrier but in whether it can convert “follicular drug concentration” into “patient-visible hair density” in an affordable manner. Current research urgently needs to break free from “technological over-competition” and establish a holistic mindset encompassing “clinical problem identification, mechanism exploration, carrier adaptation, and translational implementation”. Only then can transdermal delivery truly bridge the gap between “theoretical efficiency” and “clinical efficacy”.

## Data Availability

Data sharing is not applicable to this article as no new data were created or analyzed in this review.
